# Three-year results of a randomized clinical trial comparing retrorectus synthetic mesh and biological mesh for incisional hernia prevention following loop ileostomy closure (Preloop trial)

**DOI:** 10.1093/bjsopen/zraf058

**Published:** 2025-05-17

**Authors:** Elisa J Mäkäräinen, Heikki T Wiik, Jyrki A O Kössi, Tarja M Pinta, Leena-Mari J Mäntymäki, Anne K Mattila, Pasi P Ohtonen, Tero T Rautio

**Affiliations:** Oulu University Hospital, Medical Research Centre Oulu, Oulu, Finland; Oulu University Hospital, Medical Research Centre Oulu, Oulu, Finland; Päijät-Häme Central Hospital, Gastrointestinal surgery department, Lahti, Finland; Seinäjoki Central Hospital, Gastrointestinal surgery department, Seinäjoki, Finland; Tampere University Hospital, Gastrointestinal surgery department, Tampere, Finland; Jyväskylä Central Hospital, Gastrointestinal surgery department, Jyväskylä, Finland; Oulu University Hospital, Medical Research Centre Oulu, Oulu, Finland; Oulu University Hospital, Medical Research Centre Oulu, Oulu, Finland

The incidence of incisional hernia (IH) following loop ileostomy closure has likely been underestimated until recently. However, current research indicates that IH is a common complication, with reported rates as high as 36% after loop ileostomy closure^1^. Up to half of these hernias may require surgical repair, placing an economic burden on healthcare systems that is preventable^2^.

The Preloop trial was designed in 2018 as a multicentre, randomized clinical study and aimed to compare retrorectus synthetic mesh (SM) with biological mesh (BM) in terms of safety and effectiveness after loop ileostomy closure^3^. At the time, BM was often preferred for contaminated surgical sites for fear of SM-related infectious complications. The trial was registered before initiation (NCT03445936; http://www.clinicaltrials.gov) and conducted at four hospitals in Finland between November 2018 and August 2021. Patients undergoing temporary loop ileostomy closure following anterior resection for rectal adenocarcinoma were eligible. Short-term (30-day) results of the Preloop trial demonstrated similar complication rates, length of hospital stay, and duration of operation between the groups^4^. The 10-month follow-up results showed that both meshes effectively reduced the incidence of IH, with only one computed tomography-confirmed patient with IH per group and no mesh-related complications^5^. This research letter presents the findings of the 3-year follow-up.

No external funding was received. The meshes were provided by the participating hospital districts. IH was defined as an abdominal wall gap perceptible or palpable by clinical examination. More detailed information on eligibility, randomization, procedure, and follow-up is provided in previous publications of the trial^3–5^.

Of 102 enrolled patients, 75 (73.5%) were analysed at the 3-year follow-up, with a mean (standard deviation) follow-up time of 39.2(5.8) (range 26.0–57.0) months. A clinical visit was completed with 25 of 37 patients (67.6%) in the SM group and 27 of 38 (71.1%) in the BM group (*P* = 0.352), whereas the rest were interviewed by telephone as they were asymptomatic and preferred to not travel long distances for follow-up visits. Baseline characteristics of patients at the 3-year follow-up are summarized in *Table 1*. No clinically detectable IH was observed in any study patient. One patient in the SM group had required a reoperation for liver metastases after the 10-month follow-up. None of the meshes were removed, and none of the patients had a long-term surgical site infection. Quality of life measured by RAND-36-Item Health Survey^TM^ was comparable between the groups. Detailed data on baseline demographics, operative times, and previous outcomes are available in previous publications (4,5). Neither patient who had computed tomography-confirmed IH at the 10-month follow-up had developed a symptomatic IH^5^.

**Table 1:** Patient characteristics at 3 years’ follow-up

	SM (*n* = 37)	BM (*n* = 38)
**Sex**		
Female	11 (29.7%)	14 (36.8%)
Male	26 (70.3%)	24 (63.2%)
Age (years), mean(s.d.)	64.8(10.3)	68.8(11.3)
BMI (kg/m^2^), mean(s.d.)	26.1(4.0)	26.0(5.1)
Previous hernia	2 (5.4%)	7 (18.4%)
**ASA grade at stoma closure**		
I	6 (16.2%)	5 (13.2%)
II	20 (54.1%)	22 (57.9%)
III	11 (29.7%)	11 (28.9%)
High blood pressure	21 (56.8%)	21 (55.3%)
Diabetes mellitus	4 (10.8%)	6 (15.8%)
Adjuvant therapy after anterior resection	5 (13.5%)	7 (18.4%)
PSH at stoma closure	4 (10.8%)	6 (15.8%)
Width of mesh (cm), mean(s.d.)	7.2(1.1)	5.0(0.0)
Length of follow-up (months), mean(s.d.)	39.0(6.0)	39.3(5.7)

SM, synthetic mesh; BM, biological mesh; s.d., standard deviation; BMI, body mass index; ASA, American Society of Anesthesiologists; PSH, parastomal hernia.

This study demonstrated that both retrorectus SM and BM effectively prevented clinically significant IH. There were no mesh- or surgical site-related complications. The present results are clinically significant given the high IH rates reported after loop ileostomy closure without a mesh^1^.

In conclusion, both SM and BM appear to be safe and effective, but long-term follow-up is needed to confirm these findings.

## Data Availability

The data that support the findings of this study are available from the corresponding author upon reasonable request.
